# Pseudoginsengenin DQ exerts antitumour activity against hypopharyngeal cancer cells by targeting the HIF-1α-GLUT1 pathway

**DOI:** 10.1186/s12935-021-02080-x

**Published:** 2021-07-19

**Authors:** Sanchun Wang, Yu Cai, Qingjie Feng, Jing Gao, Bo Teng

**Affiliations:** 1 Department of Otorhinolaryngology Head and Neck Surgery, The Second Hospital of Jilin University, Changchun, China; 2 State Key Laboratory of Electroanalytical Chemistry, Changchun Institute of Applied Chemistry, Chinese Academy of Sciences, Changchun, China

**Keywords:** Pseudoginsengenin DQ, Hypopharyngeal cancer, Antitumour, HIF-1α-GLUT1, dSTORM

## Abstract

**Background:**

Ginsenosides have been reported to possess a variety of biological activities. Synthesized from the ginsenoside protopanaxadiol (PPD), the octanone pseudoginsengenin DQ (PDQ) may have robust pharmacological effects as a secondary ginsenoside. Nevertheless, its antitumour activity and molecular mechanism against hypopharyngeal cancer cells remain unclear.

**Methods:**

Cell Counting Kit8 assays, cell cycle assays and cell apoptosis assays were conducted to assess FaDu cell proliferation, cell phase and apoptosis. The interactions between PDQ and HIF-1α were investigated by a molecular docking study. The expression of HIF-1α, GLUT1, and apoptosis-related proteins was detected by Western blotting, direct stochastic optical reconstruction microscopy (dSTORM) and qRT-PCR. A glucose uptake assay was used to assess the glucose uptake capacity of FaDu cells.

**Results:**

PDQ suppressed proliferation, reduced glucose uptake, and induced cell cycle arrest and apoptosis in FaDu cells. A molecular docking study demonstrated that PDQ could interact with the active site of HIF-1α. PDQ decreased the expression and mRNA levels of HIF-1α and its downstream factor GLUT1. Moreover, the dSTORM results showed that PDQ reduced GLUT1 expression on the cell membrane and inhibited GLUT1 clustering.

**Conclusion:**

Our work showed that the antitumour effect of PDQ was related to the downregulation of the HIF-1α-GLUT1 pathway, suggesting that PDQ could be a potential therapeutic agent for hypopharyngeal cancer treatment.

**Supplementary Information:**

The online version contains supplementary material available at 10.1186/s12935-021-02080-x.

## Introduction

Hypopharyngeal carcinoma is one of the most challenging head and neck malignancies to treat and still has one of the worst prognoses of head and neck malignant tumours [1]. The 5-year overall survival rate is only 30%–35% [2]. Surgery followed by radiotherapy has been the traditional treatment for advanced-stage patients [3], but total laryngectomy will cause them to lose voice function. Although the strategy of organ preservation with chemotherapy can achieve a survival rate similar to that of surgery plus radiotherapy and retain laryngeal function [4], chemotherapy has some acute and late toxicities. Therefore, it is indispensable to seek more effective and novel natural drugs with few side effects to treat hypopharyngeal cancer.

Ginseng has long been used as a general tonic to strengthen immunity and prolong life in traditional Chinese medicine [5]. As the main effective constituents of ginseng, ginsenosides possess a wide spectrum of pharmaceutical activities, including central nervous regulation, immune function enhancement, cardiovascular health protection, and antiaging and antitumour activities [6]. Hypoxia-inducible factor 1 (HIF-1) is an essential transcription factor contributing to cellular oxygen sensing and adaptation to hypoxia. The enzymes of glucose metabolism, including glucose transporter 1 (GLUT1), are regulated by HIF-1, and the expression of these genes regulated by HIF1 alters intracellular biological functions such as glucose uptake and energy production [7, 8], which is associated with cancer cell proliferation and poor prognosis in tumours [9, 10]. Studies have shown that ginsenoside can inhibit the growth of liver or lung cancers by inhibiting hypoxia-inducible factor-1α (HIF-1α)-mediated glucose metabolism [11, 12]. With the deepening of research, it has been found that secondary ginsenosides and aglycones produced by the degradation of ginsenosides have stronger pharmacological effects [13]. Pseudoginsengenin DQ (PDQ) is a kind of octylon ginsenoside synthesized from protopanaxadiol saponins by oxidation and cyclization under acidic conditions [14]. Recent studies have demonstrated that PDQ can be used to improve aconitine-induced arrhythmia or cisplatin-induced acute kidney injury [15, 16]. However, its antitumour activity against hypopharyngeal carcinoma cells and the underlying mechanism have rarely been investigated.

Previous studies usually used classical methods such as Western blotting, real-time RT-PCR and flow cytometry to investigate topics similar to those described above. These methods are based on the average of the ensemble level of related molecules. To reflect the spatial distribution and structural arrangement of signal proteins at the single-molecule level, one of the superresolution imaging techniques, direct stochastic optical reconstruction microscopy (dSTORM) [17], needs to be utilized. dSTORM relies on fluorophores that can be switched between bright-on and a dark-off states. Only a few molecules are randomly excited to the bright-on state, and their positions are recorded. By repeating this process, a reconstructed superresolution image is finally obtained by accumulating the precise locations of each detected molecule. This approach allows the direct observation of protein distribution with a resolution of decades of nanometres [18, 19], and reveals the localization or arrangement of proteins [20]. Thus, biochemical methods and dSTORM are complementary to each other, and combining them is beneficial for acquiring both overall and single-molecule information on PDQ’s antitumour mechanism.

In this work, we investigated the antitumour effects of PDQ on human hypopharyngeal carcinoma FaDu cells. PDQ was found to suppress cell proliferation, induce cell apoptosis and trigger cell cycle arrest. Furthermore, molecular docking was performed to identify HIF-1α as the antitumour target of PDQ in hypopharyngeal carcinoma. Ultimately, the underlying molecular mechanism of PDQ’s antitumour effect, through the inhibition of the HIF-1α-GLUT1 pathway, was elucidated by classical biotechnological techniques and superresolution fluorescence microscopy.

## Materials and methods

### Cell culture

The FaDu cell line was purchased from the Shanghai Institute of Biological Sciences and cultured in high glucose Dulbecco’s modified Eagle’s medium (DMEM; Gibco) supplemented with 10% foetal bovine serum (FBS; Gibco) and 100 μg/ml penicillin/streptomycin (P/S; Invitrogen). Cells were maintained at 37 °C with 5% CO_2_.

### Cellular cytotoxicity assay for PDQ

PDQ was obtained from the School of Pharmaceutical Sciences of Jilin University, Changchun, China. FaDu cells in the logarithmic growth phase were seeded in 96-well plates at 5000 cells/well and cultured overnight. Then, the cells were treated with different concentrations of PDQ (40, 60, 80, 100, 120, 140, 160 and 180 μmol/L) for 24 h, 48 h and 72 h. Cells treated with the same amount of anhydrous ethanol (PDQ = 0 μmol/L) were used as controls. The final concentration of ethanol was 0.1%. Five parallel samples were used for each concentration. After that, 10 μL of Cell Counting Kit 8 solution (CCK8; Beyotime) was added to every well, and the cells were incubated for 2 h at 37 °C with 5% CO_2_. Finally, the absorbance at 450 nm of every well was read by a Bio-Rad microplate reader (model 630; Hercules, CA, USA).

### Safety detection of PDQ in vivo

Ten female BALB/c mice (18–22 g) were purchased from the Animal Centre of Norman Bethune Medical College of Jilin University (Changchun, China). All animals were maintained under standardized laboratory conditions (12 h light/dark cycle beginning at 08:00 a.m., temperature 22–25 °C, relative humidity 50–70%) with free access to food and water. The mice were randomly divided into the control and PDQ groups (5 mice/group). Mice in PDQ group were gavaged with 40 mg/kg PDQ (suspended in 0.05% carboxymethylcellulose sodium) once, while mice in the control group were treated with an equal amount of carboxymethylcellulose sodium. All mouse general clinical conditions were observed daily for a 2-week follow-up period. Mice were sacrificed by cervical vertebra dislocation at day 14. Whole blood was collected from the orbit, and the serum was separated from the blood by centrifugation (3500 rpm, 15 min, 4 °C). Haematopoietic and biochemical analyses were performed by an automatic haematology analyser (HC2200, Meiyilinm, China). The heart, lungs, liver, spleens and kidneys were rapidly collected and cut into 4–5 μm-thick sections, embedded in paraffin, stained with haematoxylin–eosin and examined with a Nikon TE 2000 fluorescence microscope (Nikon, Japan). All animals in this study were handled according to a protocol approved by the Institutional Animal Care and Use Committee of Jilin University (No. 2016135).

### Glucose uptake assay

Cells (3 × 10^5^ cells/well) were plated in 6-well plates. After the cells were cultured for 24 h with different concentrations of PDQ, the supernatant of the culture medium was collected to examine the concentration of glucose using a glucose assay kit (BestBio, BB-4731–1). The glucose concentration was quantified by measuring the absorbance at 505 nm with a plate reader (BioTek) and was normalized by the protein concentration of the respective samples.

### Cell cycle analysis by flow cytometry

To analyse the cell cycle, FaDu cells at the logarithmic growth phase were harvested, washed with ice-cold PBS, and then fixed with ice-cold 70%–75% ethanol at − 4 °C overnight. After that, the cells were washed with cold PBS, subsequently incubated with 100 ng/mL RNase A for 30 min at 37 °C, and then filtered through a 400-mesh screen. Next, the cells were stained with 10 μg/mL PI for 30 min at 4 °C in the dark. Finally, samples were tested by flow cytometry with FACS Diva Software (Becton Dickinson) to analyse DNA content and light scattering.

### Cell apoptosis analysis by flow cytometry and TUNEL

To analyse cell apoptosis, FaDu cells were plated in 6-well plates and treated with different concentrations of PDQ (60, 100 and 140 μmol/L) or with the same amount of anhydrous ethanol. After 24 h, cells were harvested and labelled with an Annexin V-FITC-PI Apoptosis Detection Kit (BD Biosciences, Beijing, China) according to the manufacturer’s instructions and analysed by flow cytometry (Becton Dickinson).

Moreover, immunofluorescence staining of TUNEL was also performed to detect cell apoptosis. Cells were cultured to high confluence of 80%–90% and treated with different concentrations of PDQ (0, 60, 100, and 140 μmol/L). After 24 h of incubation, the cells were fixed with 4% paraformaldehyde for 30 min and permeabilized with 0.5% Triton X-100 for 10 min. Next, cells were incubated with TUNEL (Promega Corp., Madison, WI, USA) at 37 °C for 30 min, followed by incubation with Alexa Fluor 488-conjugated goat anti-mouse IgG (Cell Signaling Technology) for 1 h. Finally, the samples were observed by a Zeiss 510 Meta laser scanning confocal microscope (Zeiss). The exposure time of all pictures was 200 ms.

### Molecular docking

Schrödinger Suites (2015) software was used to perform molecular docking between PDQ and protein receptors. The crystal structures of proteins were obtained from the Protein Data Bank. The three-dimensional (3D) structure of PDQ was drawn via ‘Maestro Elements’ in the software, and its bond angle and order were assigned by ‘Ligand Preparation’. Protein receptors and PDQ were docked with ‘Glide Docking’, and their docking results were visualized through the PyMOL Molecular Graphics System.

### Quantitative real-time RT-PCR

FaDu cells were cultured in 96-well plates and treated with different concentrations of PDQ (60, 100 and 140 μmol/L) or with the same amount of anhydrous ethanol. After incubation for 24 h, total RNA was extracted using TRIzol reagent (Invitrogen, USA). cDNA was synthesized by reverse transcription ReverTra Ace® qPCR RT Master Mix with gDNA Remover (Toyobo Co., Ltd.) and subjected to real-time PCR with gene-specific primers in the presence of Cybergreen (Applied Biosystems). Experiments were performed at least three times with duplicate replicates. The relative abundance of mRNA was calculated by normalization to GAPDH. The primer pairs used here (purchased from Genecopoeia Co., Ltd.) were as follows: GAPDH: forward 5′- TTCTTTTGCGTCGCCAGCCGAG—3′, reverse 5′- CCAGGCGCCCAATACGACCAAA—3′; GLUT1: forward 5′- CTGGCATCAACGCTGTCTTC—3′, reverse 5′- GCCTATGAGGTGCAGGGTC—3′; HIF-1α: forward 5′- AGACAAAGTTCACCTGAGCC—3′, reverse 5′- GGGAGCTAACATCTCCAAGTCT—3′.

### Western blotting

FaDu cells were treated with different concentrations of PDQ (60, 100 and 140 μmol/L) or with the same amount of anhydrous ethanol. After incubation for 24 h, cells were harvested and subjected to SDS-PAGE, after which the proteins were identified per the antibody manufacturer’s instructions (BD Biosciences). The following primary antibodies were used: anti-HIF-1α (1:200 dilution, Santa Cruz, sc-13515), anti-GLUT1 (1:200 dilution, Santa Cruz, sc-377228), anti-Bcl-2 (1:1000 dilution; Bioss, bsm-52304R), anti-Bax (1:1000 dilution; Bioss, bsm-52316R), anti-Caspase9 (1:800 dilution; Proteintech, 10380–1-AP), anti-Caspase3 (1:1000 dilution; Proteintech, 19677–1-AP), and GAPDH (1:10,000 dilution; Abcam, Inc.). Following incubation with the corresponding secondary antibodies, the signals were developed using an Amersham ECL Plus Western blotting Detection System (GE Healthcare). Data are presented as relative protein levels normalized to GAPDH, and the ratio of control samples was taken as 100%.

### dSTORM imaging

Cells were passaged on a precleaned standard microscope slide (22 mm × 22 mm, Fisher) in the dish and treated with 100 μM PDQ or left untreated. After 24 h, the solution was removed, and the cells were rinsed with PBS three times. Subsequently, the cells were fixed with 4% paraformaldehyde for 10 min at room temperature and blocked with 3% bovine serum albumin for 30 min. Then, the cells were incubated with anti-human GLUT1 antibodies (2 μg/ml in 3% BSA; Santa Cruz, sc-377228) overnight at 4 °C and washed with PBS. Finally, cells were stained with Alexa Fluor 647 goat anti-mouse IgG antibodies (2 μg/ml in 3% BSA; Invitrogen, A-21235) for 1 h. Before imaging, 50 μl imaging buffer containing Tris (50 mM, pH 8.0), NaCl (10 mM), glucose (10% w/v), glucose oxidase (500 μg/ml; Sigma), catalase (40 μg/ml; Sigma) and β-mercaptoethanol (1% v/v; Sigma) was dropped on a large microscope slide (24 mm × 50 mm, Fisher), and the small slide on which the cells were seeded was covered by the large slide and sealed with nail polish.

dSTORM imaging was performed on a Nikon Ti-E microscope with a 100 × 1.49 NA TIRF lens (Nikon, Japan). The sample was illuminated in total internal reflection fluorescence (TIRF) mode. A 640 nm laser was used to excite Alexa Fluor 647 fluorophores, and a 405 nm laser was used to increase the number of on-state fluorophores by carefully controlling the irradiation intensity (< 0.1 kW/cm^2^). All images were captured by a cooled EMCCD (Andor Ixon Ultra 888). A total of 5000 images were collected for each cell with an internal time of 25 ms between frames to reconstruct a superresolution image. TetraSpeck microspheres of 100 nm (Invitrogen) were embedded in the sample to correct x–y drift, and a focus lock was used to correct z drift.

### dSTORM data analysis

Raw dSTORM image sequences were analysed by ThunderSTORM [21], a free available plug-in in ImageJ, to obtain a reconstructed dSTORM image. To characterize the spatial distribution of GLUT1 on cell membranes, the SR-Tesseler analysis method was used as previously reported [22]. First, a file including the coordinates, intensity and sigma of localizations was loaded in the program, after which the reconstructed image was shown, and an ROI of the cell was selected for analysis (Additional file 1: Figure S1a). Second, bisectors between the two closest localizations were drawn, and the ROI was segmented into many polygons with different numbers of localizations (Additional file 1: Figure S1b). The localization density of a polygon was defined as δi1, and the average localization density of the total ROI was δ_0_. If δi_1_ > δ_0_, localizations in this polygon were picked up to create an ‘object’ (Additional file 1: Figure S1c). Similarly, the localization density of an object was set as δi_2_, and the average of all objects was δ_1_. Third, objects satisfying the requirement of δi_2_ > δ_1_ were extracted as clusters (Additional file 1: Figure S1d). Finally, the area, the number of localizations, coordinates and morphological parameters of each cluster were computed and exported.

### Statistical analysis

Data are expressed as the mean ± SD (standard deviation). All statistical analyses were performed using SPSS version 22.0 (IBM, Chicago, Illinois, USA). Group means were compared by one-way analysis of variance (ANOVA), and P values < 0.05 were considered significant in all cases. In addition, a two-tailed unpaired t-test was used for the statistical analysis of dSTORM data, and P values < 0.05 were considered significant.

## Results

### PDQ inhibits the proliferation of FaDu cells

**Fig. 1:**
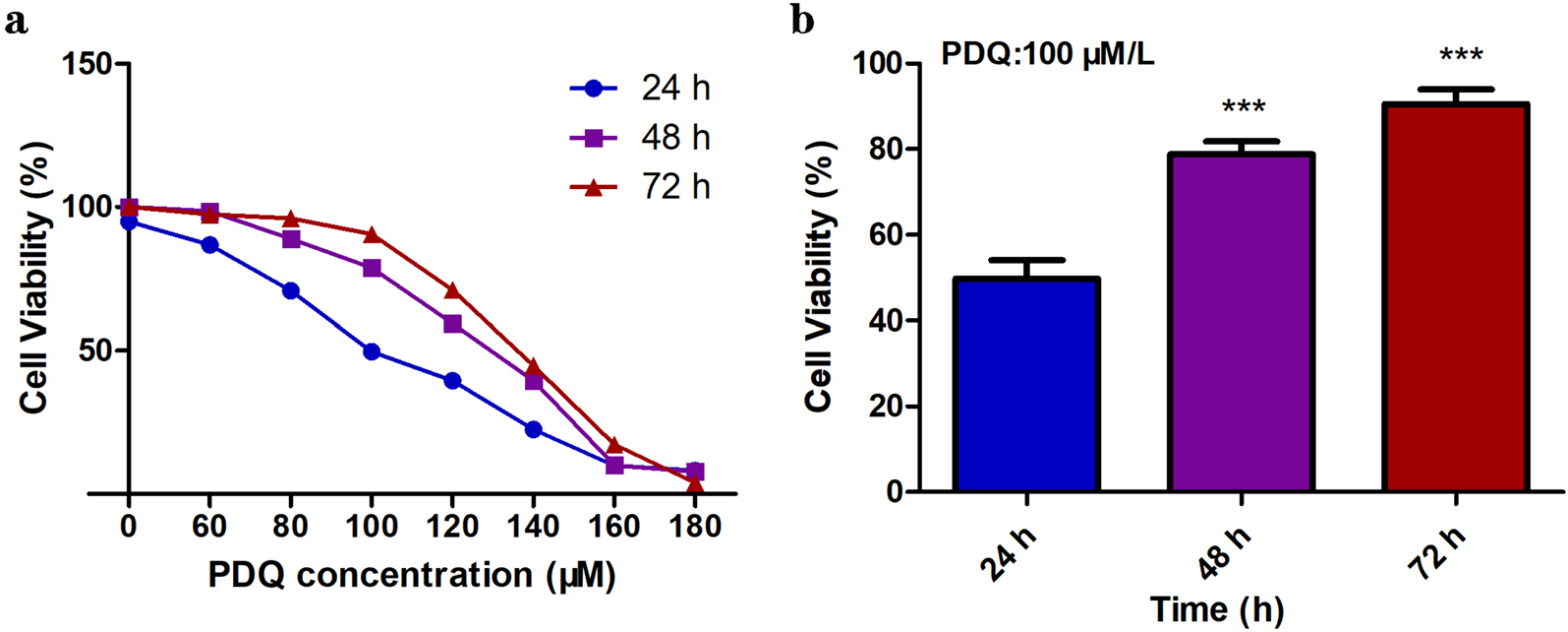
The effect of PDQ on the proliferation of FaDu cells. **a** The plots show the survival rate of cells treated with different concentration of PDQ for 24 h, 48 h, and 72 h. **b** The survival rate of cells treated with 100 μmol/L PDQ for different time. Data are shown as mean ± S.D., ***p < 0.001, compared to control, n = 3

We first carried out a CCK8 assay to assess the cytotoxicity of PDQ in FaDu cells. As shown in Fig. 1, cells were exposed to different concentrations of PDQ for 24 h, 48 h and 72 h. The plots indicated that the cell survival rate was the lowest and that the IC50 value (100 μmol/L) was minimal after exposure for 24 h. For 48 h and 72 h, the survival rate increased slightly. Moreover, PDQ significantly inhibited the proliferation of FaDu cells when the concentration of PDQ was higher than 60 μmol/L, and the inhibition was dose-dependent. The results suggested that PDQ inhibited the proliferation of hypopharyngeal cancer cells in a dose-dependent but time-independent manner. Accordingly, we used the effective PDQ concentration in the range of 60–140 μmol/L and 24 h of treatment in subsequent experiments.

To exclude the possibility that the safety of PDQ and its systematic effect may lead to cell growth inhibition, we performed an in vivo experiment. No mice died during the 14-day follow-up period. The haemogram assay indicated that the white blood cell (WBC), red blood cell (RBC), lymphocyte (LYM) and monocyte (MONO) counts were not significantly different between the PDQ and control groups. The levels of the serum biochemical markers ALT, AST, BUN and CRE were not significantly different between the two groups (Additional file 1: Table S1). To evaluate the effect of PDQ on major organs, we analysed the pathological patterns of the heart, liver, spleen, lung and kidney, and no major histological changes were observed in the PDQ group compared to the control group (Additional file 1: Figure S2). The results verified that PDQ had no side effects on animals.

### PDQ triggers cell cycle arrest in cancer cells

**Fig. 2:**
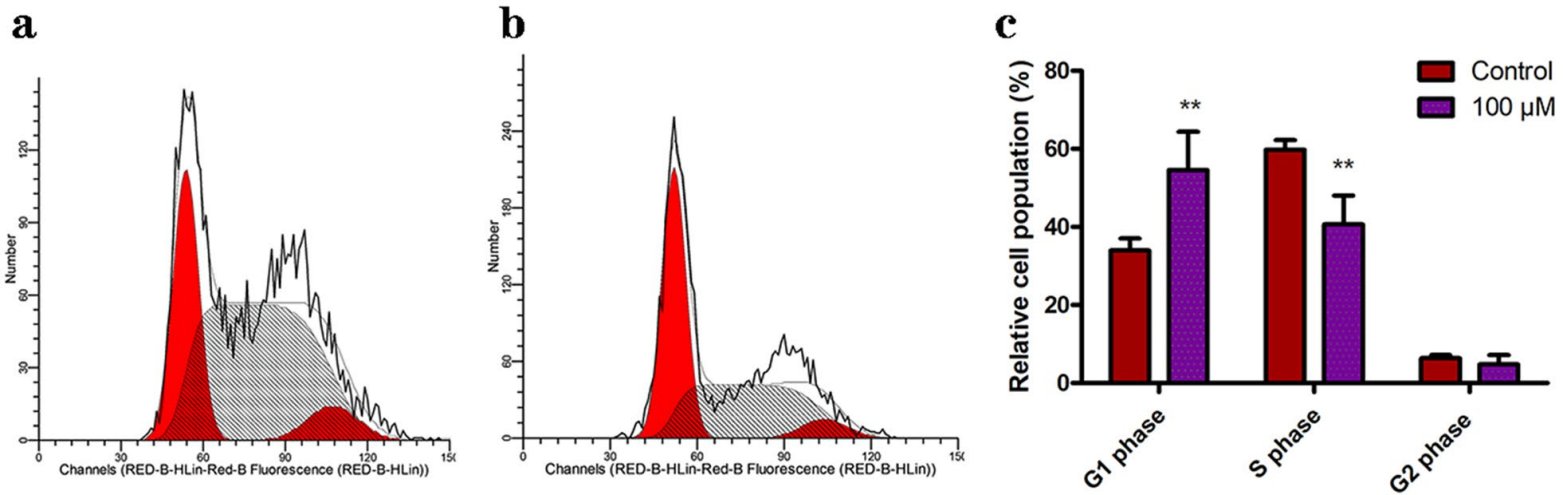
Flow cytometric detection of the cell cycle distribution. **a**, **b** The distribution of cell cycle in control group (**a**) and 100 μmol/L PDQ-treated group (**b**). **c** The percentage of cells in each phase without or with PDQ treatment. Data are shown as mean ± S.D., **p < 0.01, compared to control, n = 3

Cell proliferation depends on the cell cycle, which is an accurately regulated process that allows the cell to duplicate and grow. Since PDQ can inhibit the proliferation of FaDu cells, we wondered whether PDQ can affect the cell cycle. To confirm this possibility, we analysed the distribution of the cell cycle by flow cytometry in control and 100 μmol/L PDQ-treated cells. The results showed that G0/G1 phase cells increased significantly, whereas S and G2/M phase cells were present at a lower proportion after PDQ treatment (Fig. 2). This finding validated our hypothesis that PDQ caused cell cycle arrest in the G0/G1 phase.

### PDQ induces the apoptosis of FaDu cells

**Fig. 3:**
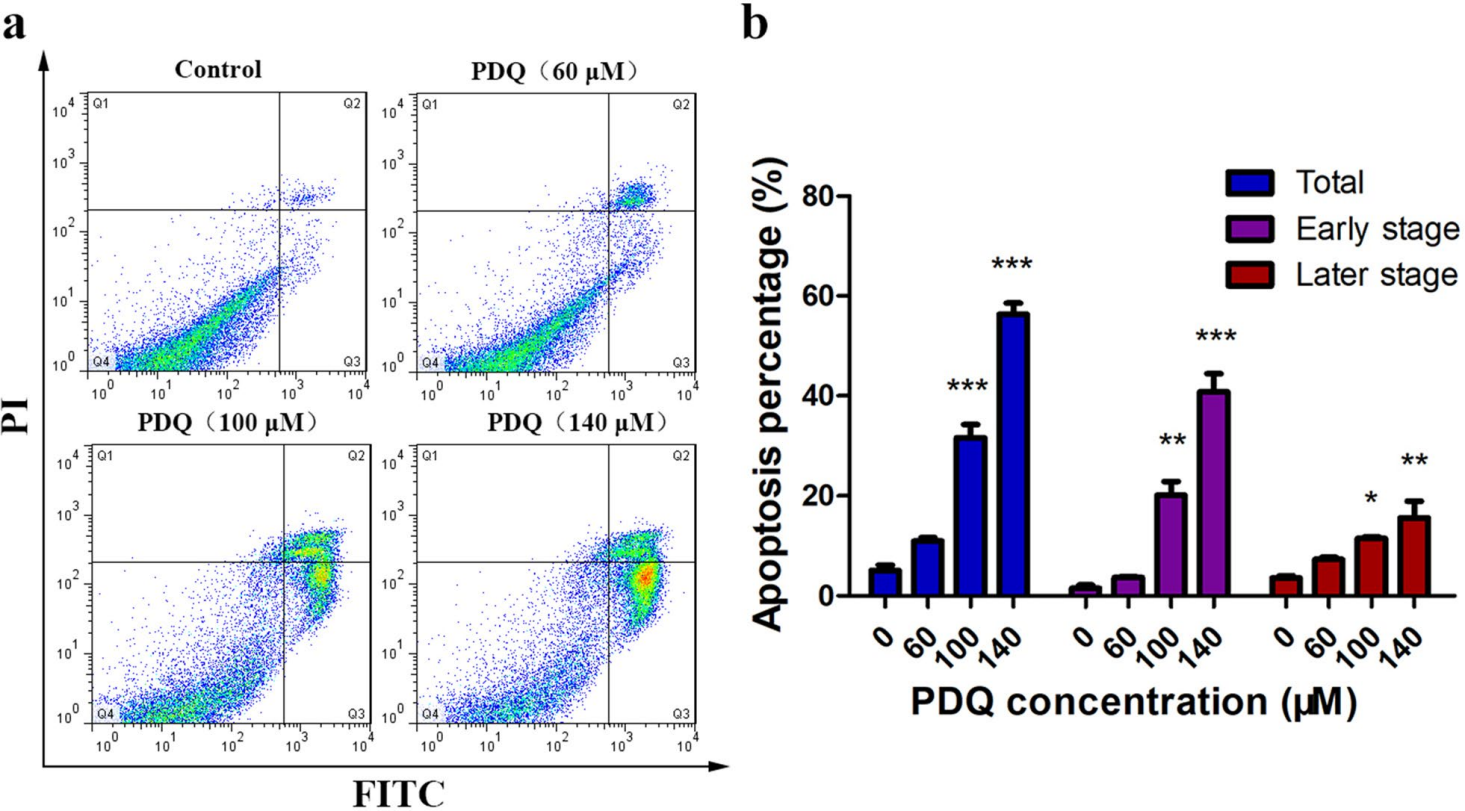
Flow cytometric analysis of the apoptosis of FaDu cells. **a** Annexin V-FITC and PI double staining to test the cell apoptosis without or with the treatment of 60, 100, and 140 μmol/L PDQ for 24 h. The Q1-Q4 quadrant represent necrotic cells, late apoptotic cells, early apoptotic cells and viable cells, respectively. **b** The percentage of total, early and late apoptotic cells treated with the different concentration of PDQ. Data are shown as mean ± S.D., *p < 0.05, **p < 0.01, ***p < 0.001, compared to control, n = 3

To evaluate the effects of PDQ on apoptosis, annexin V-FITC and PI double staining was investigated in PDQ-treated FaDu cells by flow cytometry. The results revealed that the proportion of both early and late apoptotic cells remarkably increased in a dose-dependent manner (Fig. 3a). Specifically, the rate of apoptotic cells increased from 5.06 ± 1.94% in the control group to 31.67 ± 4.5% in the cells treated with 100 μmol/L PDQ for 24 h and 56.37 ± 3.80% in the cells treated with 140 μmol/L PDQ for 24 h (Fig. 3b).

**Fig. 4:**
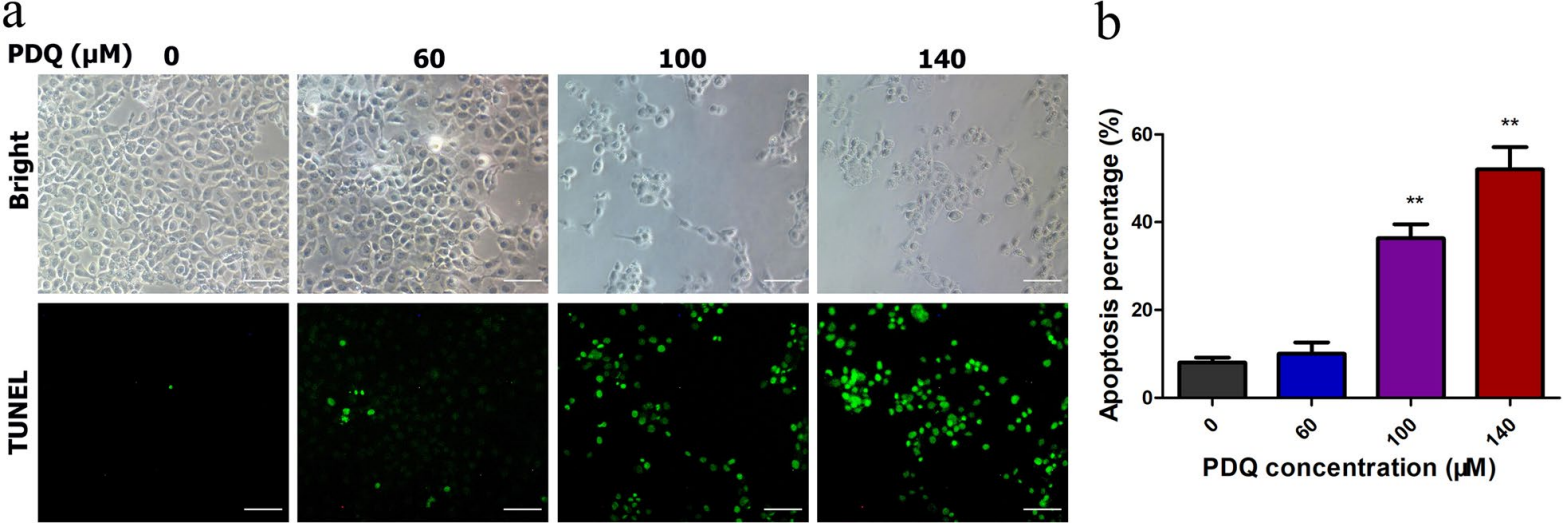
TUNEL analysis of the apoptosis of FaDu cells. **a** Confocal images of cells under the bright field and TUNEL staining without or with the treatment of different concentration of PDQ. **b** The percentage of apoptotic cells from TUNEL analysis. Data are shown as mean ± S.D., **p < 0.01, compared to control, n = 3. Scale bars are 50 μm

Subsequently, immunofluorescence analysis with TUNEL staining was used to assess the morphology of apoptotic FaDu cells and further support the results of the apoptosis effect of PDQ. Apoptotic cells usually exhibit nuclear condensation and DNA fragmentation, and the fragmented DNA in nuclei can be observed as green fluorescence signals under fluorescence microscopy after TUNEL staining. As illustrated in Fig. 4a, cells with a TUNEL-positive signal increased gradually as the PDQ concentration increased. In particular, for treatment with 100 or 140 μmol/L PDQ for 24 h, the percentage of apoptotic cells was 50.67 ± 3.05% and 71.00 ± 1.73%, respectively (Fig. 4b), which was much higher than that of the control group (3.66 ± 1.15%). Collectively, both flow cytometry and TUNEL assays demonstrated that PDQ significantly triggered the apoptosis of FaDu cells in a dose-dependent manner.

**Fig. 5:**
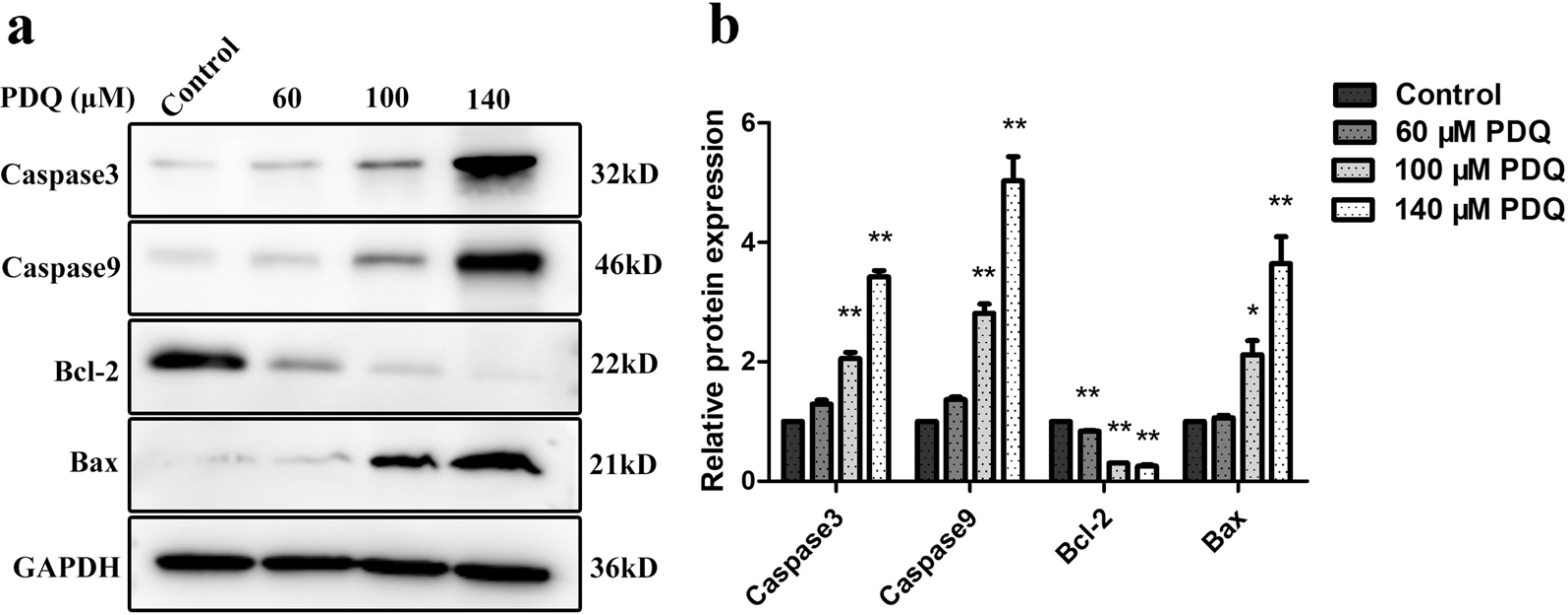
Western blot analysis of apoptosis-associated proteins. **a** The expression of apoptosis-associated proteins with the different concentration of PDQ treatment. **b** The relative protein expression is calculated after correction for the GAPDH loading control. Data are shown as mean ± S.D., *p < 0.05, **p < 0.01, compared to control, n = 3

To determine the effects of PDQ treatment on the expression of apoptosis-related proteins, the levels of Bcl-2, Bax, Caspase9, and Caspase3 were examined by Western blot assays. PDQ treatment significantly increased the expression levels of the apoptotic protein Bax and suppressed the expression of the antiapoptotic protein Bcl-2 in a dose-dependent manner. Moreover, PDQ upregulated the expression of the apoptosis-associated enzymes Caspase9 and Caspase3 in a concentration-dependent manner (Fig. 5a, b). These results demonstrated that PDQ-induced apoptosis was achieved by the regulation of apoptosis-related proteins.

### Molecular docking between PDQ and HIF-1α

**Fig. 6:**
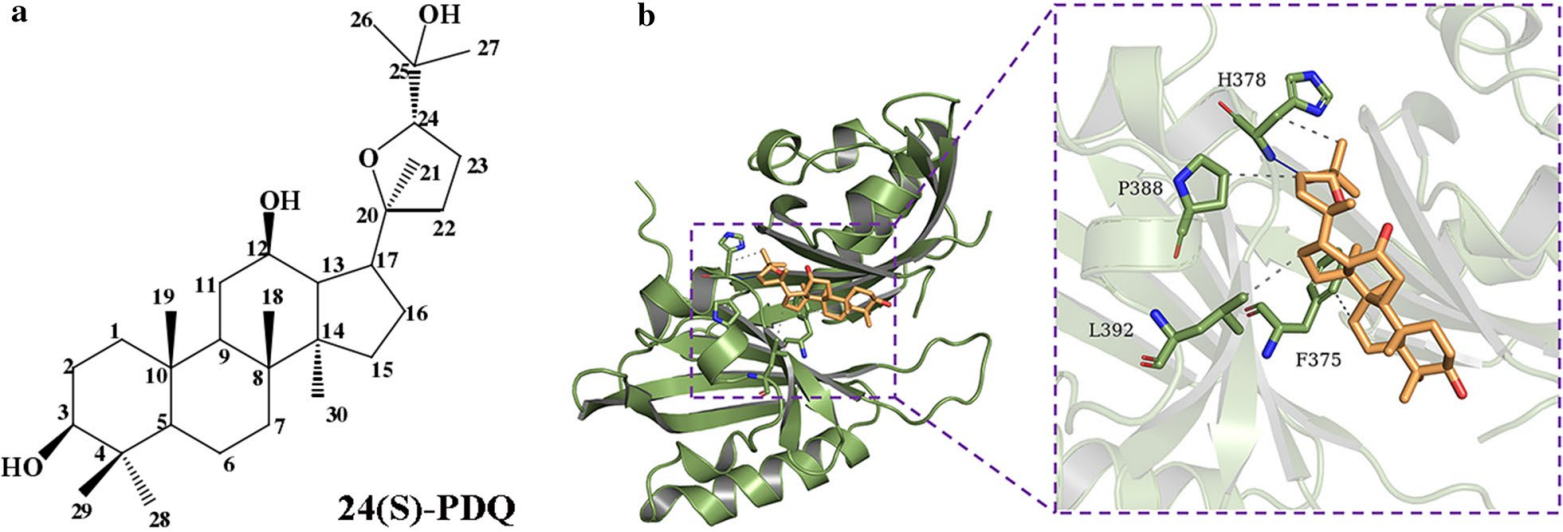
Molecular docking of ginsenoside PDQ and HIF-1α. **a** The chemical structure of 24 (S)-PDQ. **b** Molecular docking of PDQ in the active site of HIF-1α. The C-25 hydroxyl group in PDQ structure forms a hydrogen bond with His378 residue of HIF-1α (PDB ID: 4H6J). The docking score is − 7.3

PDQ can suppress cell proliferation, lead to cell cycle arrest and induce cell apoptosis. Since we verified its antitumour activity against hypopharyngeal cancer cells, we next wanted to explore the underlying mechanisms of action. To determine whether PDQ is associated with HIF-1α or GLUT1, we performed a molecular docking study. The chemical structure of PDQ is shown in Fig. 6a. After the precise docking of PDQ with these two proteins, we found that PDQ had a high docking score with HIF-1α, which was − 7.3. As shown in Fig. 6b, the C-25 hydroxyl group of PDQ interacted with residue His378 through a hydrogen bond in the active site of HIF-1α. The above results indicated that PDQ has a high affinity for HIF-1α and displays a key molecular docking interaction with contiguous amino acids in the active site of HIF-1α, which hinted that the antitumour effect of PDQ might be related to the HIF-1α signalling pathway.

### PDQ downregulates HIF-1α-GLUT1 pathway

**Fig. 7:**
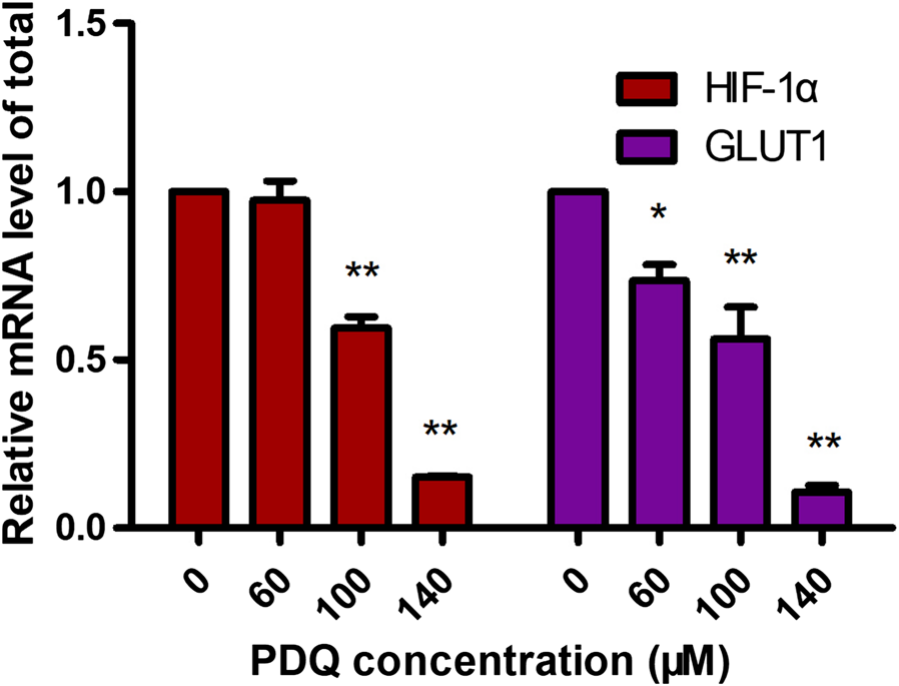
Quantitative RT-PCR analysis of HIF-1α and GLUT1 transcription with PDQ treatment. The relative levels of their mRNA under the different concentration of PDQ are displayed in a histogram. Data are shown as mean ± S.D., *p < 0.05, **p < 0.01, ***p < 0.001, compared to control, n = 3

HIF-1α is a ubiquitously expressed maternal regulator of genes that allows adaptation to hypoxic conditions [23, 24]. Its target genes include VEGF, erythropoietin, LDHA, GLUT1 and other factors critical to vascularization, metabolic regulation, cell multiplication and survival [25]. As HIF-1α and GLUT1 are considered intrinsic hypoxia markers, we selected GLUT1, one of the important HIF-1α downstream effectors, for further study. To further verify that PDQ is interrelated with the HIF-1α-GLUT1 pathway in FaDu cells, we analysed the mRNA expression of HIF-1α and GLUT1 using a qRT-PCR assay. FaDu cells were treated with different concentrations of PDQ (0, 60, 100 and 140 μmol/L). Figure 7 reveals that PDQ significantly inhibited HIF-1α expression at the mRNA level in a concentration-dependent fashion. The mRNA level of GLUT1 was markedly reduced when the concentration of PDQ was increased to 100 μmol/L or 140 μmol/L. The results indicated that high-concentration PDQ treatment significantly decreased the mRNA expression of HIF-1α and GLUT1.

**Fig. 8:**
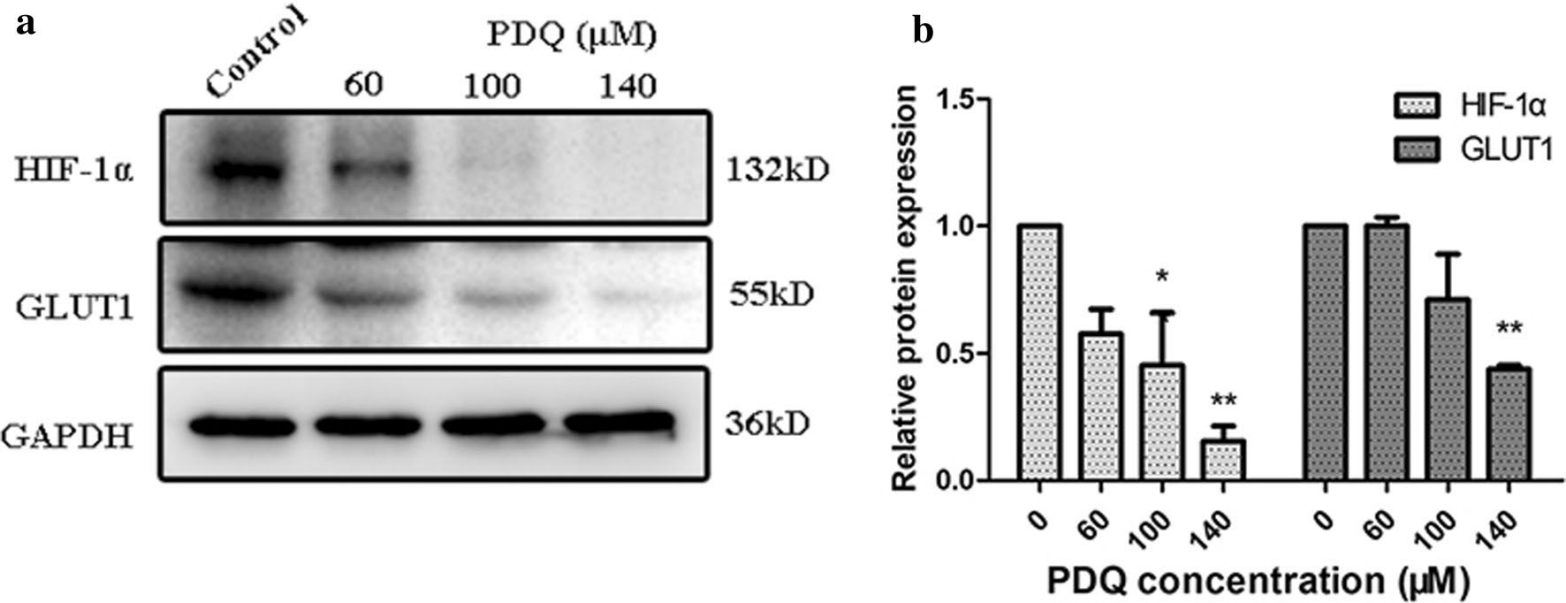
**a** Western blot analysis of the expression levels of HIF-1α and GLUT1 with PDQ treatment and quantitation of the data by Image J. **b** The relative protein expression is calculated after correction for the GAPDH loading control. Data are shown as mean ± S.D., *p < 0.05, **p < 0.01, compared to control, n = 3

Subsequently, to further explain the mechanism by which PDQ targets the HIF-1α-GLUT1 pathway in hypopharyngeal cancer cells, the protein levels of HIF-1α and GLUT1 were explored by Western blot assay. As observed in Fig. 8a, b, PDQ treatment resulted in a decrease in both proteins. In particular, for the 100 μmol/L and 140 μmol/L PDQ groups, HIF-1α expression decreased to approximately 0.47 and 0.16 times that of the control group, and GLUT1 expression decreased to 0.75 and 0.45 times that of the control group, respectively, which was in agreement with the results of RT-PCR analysis.

**Fig. 9:**
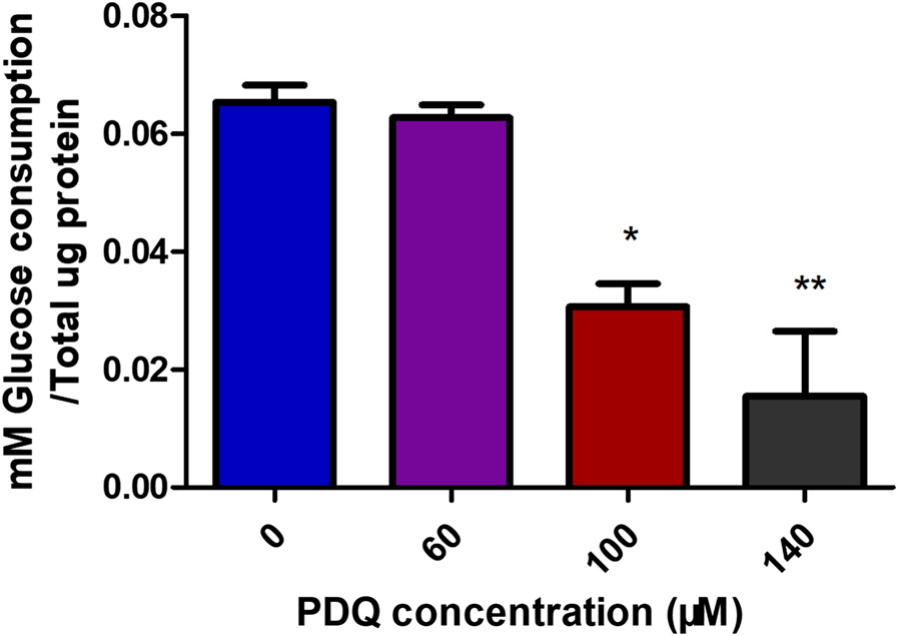
Glucose uptake analysis of relative levels of glucose consumption under the different concentration of PDQ. Data are shown as mean ± S.D., *p < 0.05, **p < 0.01, compared to control, n = 3

Since the expression levels of HIF-1α and GLUT1 were decreased by PDQ treatment and affected the energy supply of cancer cells, we investigated the glucose uptake of FaDu cells in response to PDQ treatment. The cells were treated with different PDQ concentrations for 24 h. Compared to the control group, PDQ significantly inhibited glucose uptake when the concentration of PDQ increased to 100 μmol/L or 140 μmol/L (Fig. 9). This demonstrated that PDQ inhibited glucose uptake significantly and suppressed cell proliferation by reducing the energy supply. Taken together, the above results demonstrated that PDQ downregulated the HIF-1α-GLUT1 signalling pathway, which may contribute to the antitumour activity of PDQ.

**Fig. 10:**
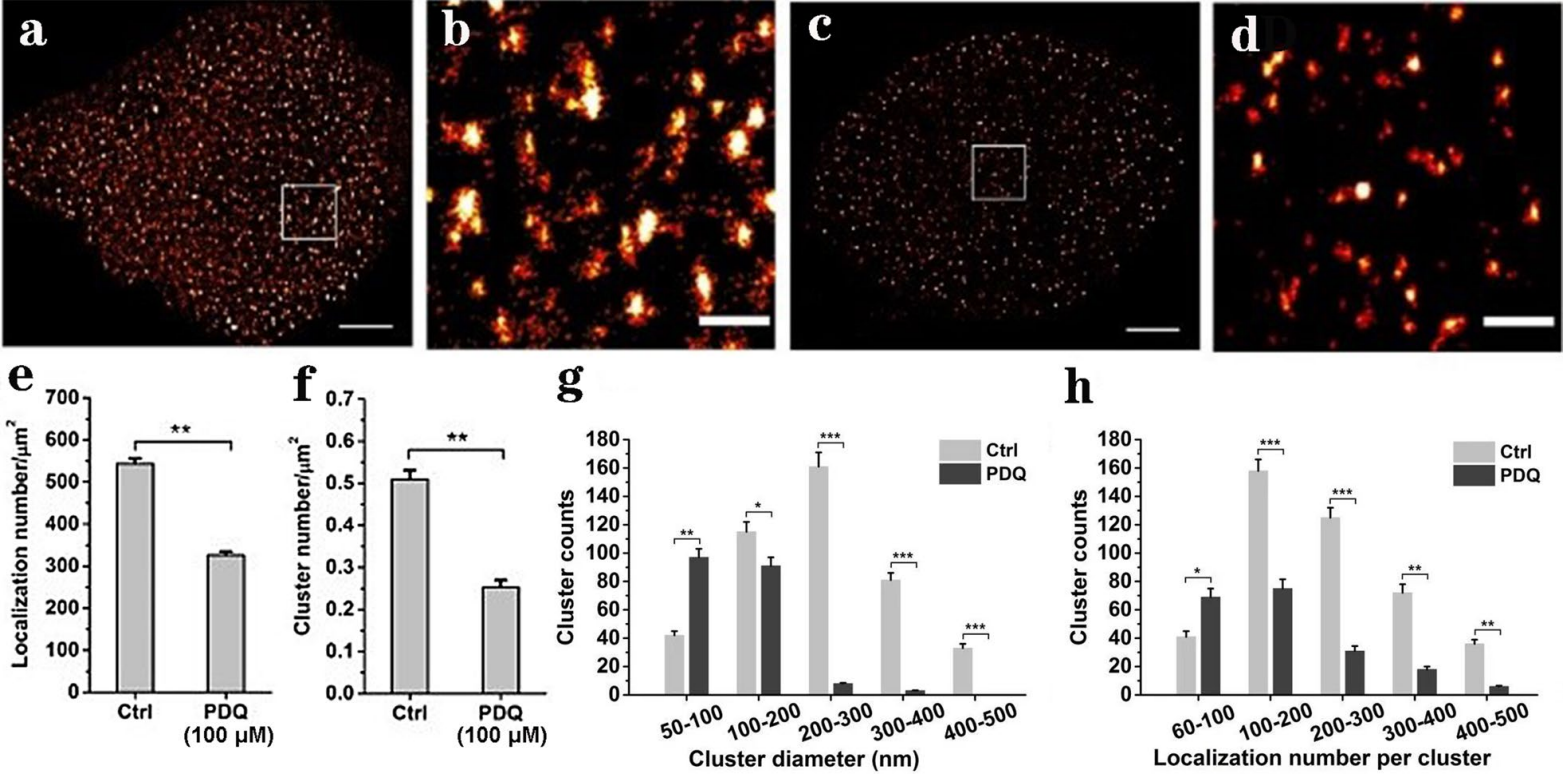
PDQ affected the distribution of GLUT1 and weakened its clustering on the membrane. **a**, **c** dSTORM imaging of GLUT1 on FaDu cell membrane without treatment (**a**) or with 100 μM PDQ treatment (**c**). **b**, **d** Magnified regions of GLUT1 corresponding to the white box in (**a**) and (**c**). Scale bars, 5 μm in (**b**) and (**d**), 1 μm in (**b**) and (**d**). **e** The number of GLUT1 localizations per μm^2^ on control and PDQ-treated cell membranes. **f** The number of clusters per μm^2^. **g** The distribution of cluster diameter. **h** The distribution of localization number per cluster. Data shown in **e**–**h** are means ± s.d.. All statistics were obtained from 30 cells in five independent experiments. *P < 0.05, **P < 0.01, ***P < 0.001, two-tailed unpaired t-test

In addition to the expression level of the HIF-1α-GLUT1 pathway, to further investigate whether PDQ affects the detailed distribution of these tumour-associated proteins, we performed dSTORM imaging. As GLUT1 is a vital downstream factor of HIF-1α and is mainly distributed on the cell membrane, it is very suitable to be observed by dSTORM. Therefore, FaDu cells were treated with 100 μmol/L PDQ or left untreated, and then the labelled samples were illuminated under a 647 nm laser. As shown in Fig. 10a–d, GLUT1 on the cell membrane decreased significantly after PDQ treatment. To accurately assess the effect of PDQ on the expression levels of GLUT1, we quantitatively analysed the number of localizations (Fig. 10e), which was proportional to the amounts of proteins. The results showed that the number of localizations of GLUT1 fell from 543 ± 12 per μm^2^ to 326 ± 9 per μm^2^ with PDQ treatment, which was consistent with the Western blot assay (Fig. 8), thus again confirming the downregulation of GLUT1 by PDQ.

More intriguingly, we found that PDQ treatment changed the morphology and spatial distribution of GLUT1 on the cell membrane. To quantitatively analyse its distribution pattern, we used the SR-Tesseler method [26] to characterize the potential clusters. This method is based on Euclidean distances to divide regions and multiple thresholding of localization density to select qualified points (Additional file 1: Figure S1, see the “Materials and methods” section for details). We first analysed the cluster number of GLUT1 per unit, which decreased almost half after adding PDQ (Fig. 10f). We next extracted the information of the cluster diameter. In control cells, clusters with a diameter ranging from 200 to 300 nm occupied the greatest percentage, and clusters with diameters of 100–200 nm and 300–400 nm were the second and third most abundant, respectively (Fig. 10g). However, the clusters were very small after PDQ treatment. There were less than ten clusters with diameters of 200–400 nm, and there were no clusters with diameters greater than 400 nm. Only 50–200 nm clusters were found on the membrane. We also explored the molecular composition of clusters. There were more clusters consisting of 60–100 localizations on PDQ-treated cell membranes than on control cell membranes (Fig. 10h). For other large clusters containing more than 100 localizations, the control group had twice or even several times more than that of the PDQ-treated group. Collectively, these results indicated that PDQ regulated the redistribution of GLUT1 clusters on the membrane. It exerted an inhibitory effect on the HIF-1α-GLUT1 pathway by not only reducing the expression levels of HIF-1α and GLUT1 but also disrupting cluster formation of the downstream signalling protein GLUT1.

## Discussion

Hypopharyngeal carcinoma has one of the worst prognoses of head and neck malignant tumours. Laryngectomy can cause patients to lose voice function, and conventional radiotherapy or chemotherapy shows poor antitumour activity and significant toxicity and side effects [27, 28]. Due to its strong pharmaceutical properties and low side effects, ginseng has become a good choice in the treatment of cancer. Pseudoginsenoside DQ is an important bioactive ingredient that is synthesized from protopanaxadiol saponins. In addition to its role in the treatment of coronary heart disease, myocardial ischaemia and arrhythmia, its antitumour effect on hypopharyngeal cancer and the underlying molecular mechanism require further clarification.

In the present study, we first confirmed that PDQ can suppress the growth and viability of FaDu cells (Fig. 1). By flow cytometry, Western blotting and TUNEL assays, we next found that PDQ can induce cell cycle arrest (Fig. 2) and apoptosis (Figs. 3, 4, 5) in hypopharyngeal cancer cells. Together, we revealed that PDQ has an antitumour effect on hypopharyngeal cancer cells and that its antitumour activity is achieved by the molecular mechanisms of inhibiting proliferation and inducing apoptosis. The results were consistent with previous medical and experimental studies on ginsenosides in other cancers, such as hepatocellular carcinoma [29], cervical carcinoma [30], colorectal cancer [31], prostate cancer [32] and breast cancer [33].

Hypoxia is a common feature of malignancy, particularly solid tumours, and is able to accelerate tumour invasiveness and metastasis [34, 35]. HIF-1α is the principal mediator during the cellular adaptive response to hypoxia [23]. It regulates multiple aspects of tumourigenesis, including proliferation, differentiation, angiogenesis, energy metabolism, and metastasis [36, 37], thereby influencing the expression of target proteins, such as VEGF, erythropoietin, GLUT1, and glycolytic enzymes [25, 38]. Among these, GLUT1 can mediate glucose transport to meet the energy requirements of tumour cells. HIF-1α and GLUT1 are considered intrinsic hypoxia markers and have been studied the most in various tumours [39–43]. Their respective high expression and the correlation between their expression have been identified in many types of tumour cells and biopsy tissue samples, which may promote tumour progression and lead to a poor survival rate and prognosis [40–44]. It has been shown that ginsenoside can regulate tumour development via HIF-1 α pathway [45], including in vivo experiments [46]. To investigate whether HIF-1α or GLUT1 is the target molecule of PDQ, we performed molecular docking of PDQ with these two proteins. Molecular docking technology has been widely used to screen and evaluate the interaction between small molecules and proteins [47, 48]. The results showed that PDQ can interact with HIF-1α with a high molecular binding force (Fig. 6), suggesting that HIF-1α is very likely to be the main target of PDQ.

Since the molecular docking experiment showed the interaction between PDQ and HIF-1α, we evaluated the mRNA and protein levels of HIF-1α and GLUT1. Both their mRNA and protein levels decreased significantly after PDQ treatment (Figs. 7, 8), suggesting that PDQ’s antitumour effect may be associated with inhibiting the HIF-1α-GLUT1 pathway. This antitumour mechanism was consistent with other studies. For example, Chen et al. found that inhibiting the expression of HIF-1α decreases the GLUT1 expression level and thus causes a reduction in the tumour volume and weight of LOVO cell line xenografts [49]. Some studies have also proposed that the combined inhibition of HIF-1α and GLUT1 could be a promising cancer therapeutic strategy [50–55]. Our work demonstrated that PDQ is just one of the first choices of drugs for realizing this antitumour effect.

In addition to the overall expression level, the detailed distribution of GLUT1 on the hypopharyngeal cancer cell membrane was also revealed by dSTORM imaging (Fig. 10). With PDQ treatment, the localization number of GLUT1 decreased significantly, which was in agreement with the results of Western blot analysis. Moreover, PDQ blocked the formation of GLUT1 clusters, and even if there were still clusters on the membranes, the cluster size and the number of GLUT1 molecules in clusters decreased sharply. The clustering of GLUT1 on cancer cell membranes has been studied before [18]. These protein clusters are beneficial for the rapid response of receptors to signals and signal transmission. Thus, the weakening of GLUT1 clusters further verified that PDQ could target the HIF-1α-GLUT1 pathway by inhibiting both the expression and clustering of GLUT1.

## Conclusions

In conclusion, by combining classical biochemistry methods with superresolution fluorescence imaging, we elucidated the antitumour activities of PDQ on human hypopharyngeal cancer cells and uncovered its antitumour mechanism. PDQ can inhibit cell proliferation, induce cell apoptosis and trigger cell cycle arrest at the G0/G1 phase. More importantly, our findings indicate that HIF-1α is the target molecule of PDQ and that PDQ’s antitumour effect is associated with suppressing the HIF-1α-GLUT1 pathway by downregulating the expression of HIF-1α and GLUT1 and disrupting the clustering of GLUT1. The current work reveals the role of PDQ in blocking the energy supply of cancer cells, which provides new insights into the molecular mechanism of ginsenoside’s antitumour effect. In the future, we will explore more target molecules of PDQ in cancer cells and perform animal experiments to evaluate its therapeutic effects to further develop it as a useful drug to treat hypopharyngeal carcinoma.

## Supplementary Information

**Additional file 1:** **Figure S1.** The illustration of SR-Tesseler analysis. An original reconstructed localization map of GLUT1 was imported (a). Bisectors between the nearest localizations segmented the map into many polygons (b). By thresholding the localization density of every polygon, objects (blue) were identified (c), and clusters (red) were extracted through the second thresholding (d). Scale bars are 1 μm. **Figure S2.** H&E staining of major organs from PDQ and control groups, including the heart, liver, spleen, lung and kidney. Scale bars = 20 μm. **Table S1.** Hematological and biochemical parameters.

## Data Availability

Please contact the corresponding author for all data requests.

## Abbreviations

PDQ: Pseudoginsengenin DQ
HIF-1α: Hypoxia-inducible factor-1α
GLUT1: Glucose transporters 1
dSTORM: Direct stochastic optical reconstruction microscopy
