# Supplementary material for: Pseudoginsengenin DQ exerts antitumour activity against hypopharyngeal cancer cells by targeting the HIF-1α-GLUT1 pathway
Source: Cancer Cell Int. 2021 Jul 19;21:382. doi: 10.1186/s12935-021-02080-x (PMC8287670; doi:10.1186/s12935-021-02080-x)
Supplement: Supplementary file 1 — Additional file 1: Figure S1. The illustration of SR-Tesseler analysis. An original reconstructed localization map of GLUT1 was imported (a). Bisectors between the nearest localizations segmented the map into many polygons (b). By thresholding the localization density of every polygon, objects (blue) were identified (c), and clusters (red) were extracted through the second thresholding (d). Scale bars are 1 μm. Figure S2. H&E staining of major organs from PDQ and control groups, including the heart, liver, spleen, lung and kidney. Scale bars = 20 μm. Table S1. Hematological and biochemical parameters. [file 12935_2021_2080_MOESM1_ESM.docx]

**Supplementary**


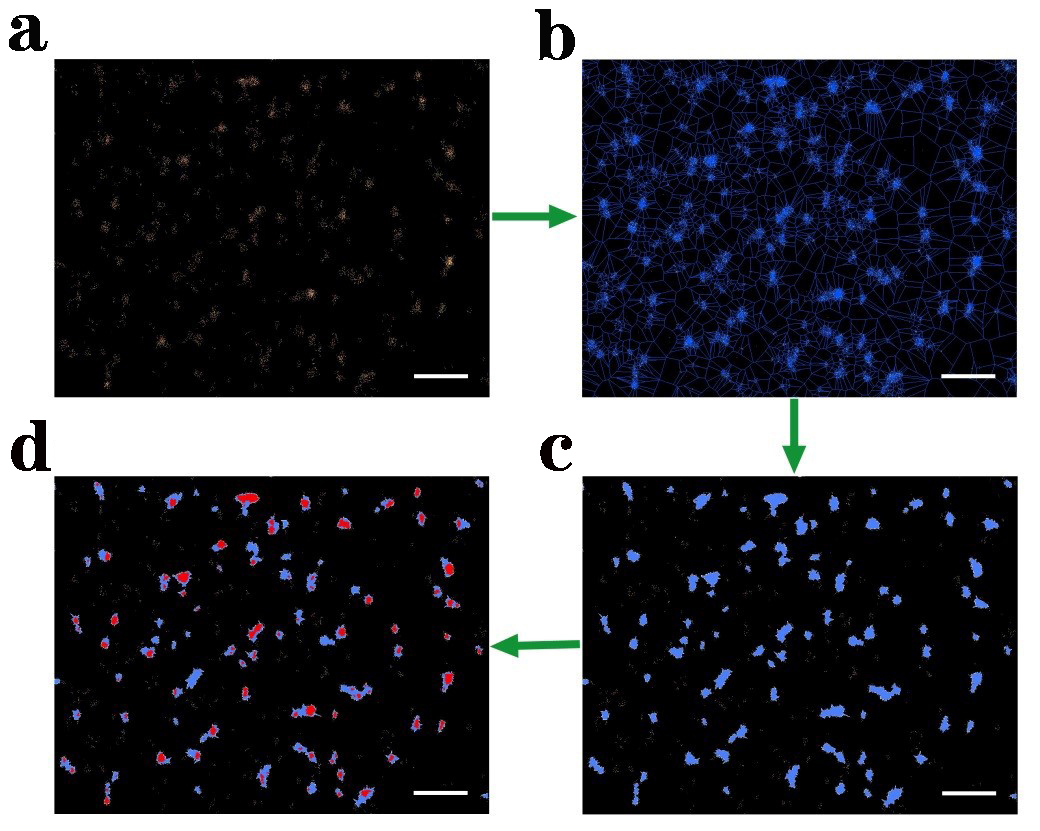


Figure S1. The illustration of SR-Tesseler analysis. An original reconstructed localization map of GLUT1 was imported (a). Bisectors between the nearest localizations segmented the map into many polygons (b). By thresholding the localization density of every polygon, objects (blue) were identified (c), and clusters (red) were extracted through the second thresholding (d). Scale bars are 1 μm.


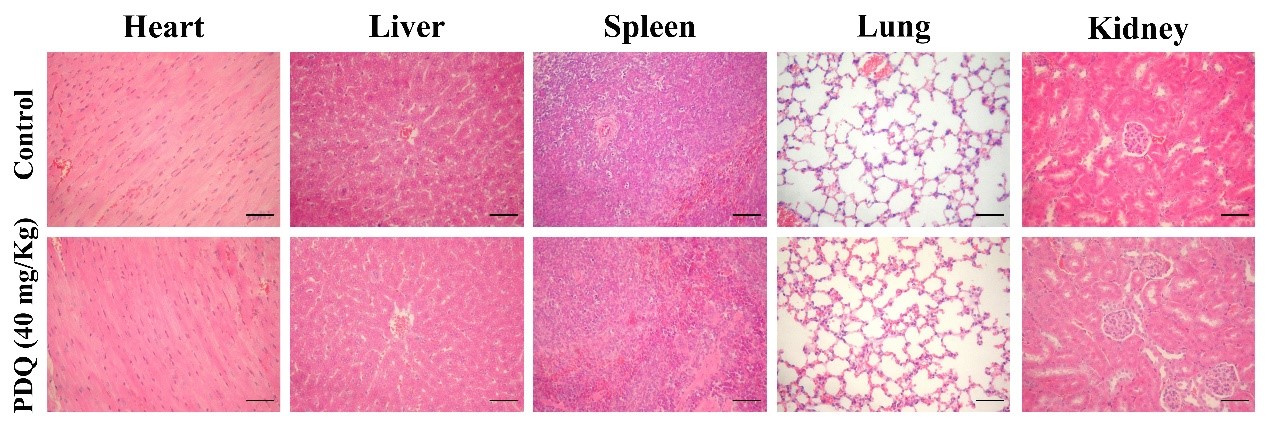


Figure S2. H&E staining of major organs from PDQ and control groups, including the heart, liver, spleen, lung and kidney. Scale bars = 20 μm.

Table S1. Hematological and biochemical parameters

| Group | Control(mean±SD) |  | PDQ（40 mg/Kg）(mean±SD) |
| --- | --- | --- | --- |
| WBC (K/μl) | 9.00±0.61 |  | 9.54±0.84 |
| RBC (M/μl) | 10.13±0.48 |  | 10.96±0.32 |
| LY (K/μl) | 7.31±0.42 |  | 7.43±0.85 |
| MONO (K/μl) | 0.72±0.02 |  | 0.83±0.04 |
| ALT (U/L) | 79.30±9.28 |  | 82.54±7.54 |
| AST (U/L) | 177.18±14.76 |  | 168.50±23.53 |
| BUN (mmol/L) | 11.09±1.86 |  | 10.52±1.08 |
| CRE (umol/L) | 34.26±9.22 |  | 30.82±4.75 |

WBC, white blood cell; RBC, red blood cell; LY, lymphocyte; MONO, monocyte; ALT, alanine aminotransferase; AST, aspartate aminotransferase; BUN, blood urea nitrogen; CRE, creatinine.
